# An Expanded Neuroimmunomodulation Axis: sCD83-Indoleamine 2,3-Dioxygenase—Kynurenine Pathway and Updates of Kynurenine Pathway in Neurologic Diseases

**DOI:** 10.3389/fimmu.2018.01363

**Published:** 2018-06-15

**Authors:** Li Bo, Tan Guojun, Guo Li

**Affiliations:** Department of Neurology, The Second Hospital of Hebei Medical University, Shijiazhuang, China

**Keywords:** soluble CD83, indoleamine 2 3-dioxygenase, kynurenine pathway, neuroimmunomodulation, neurologic disease

## Abstract

Many neurologic diseases are related to autoimmune dysfunction and a variety of molecules or reaction pathways are involved in the regulation of immune function of the nervous system. Soluble CD83 (sCD83) is the soluble form of CD83, a specific marker of mature dendritic cell, which has recently been shown to have an immunomodulatory effect. Indoleamine 2,3-dioxygenase (IDO; corresponding enzyme intrahepatic, tryptophan 2,3-dioxygenase, TDO), a rate-limiting enzyme of extrahepatic tryptophan kynurenine pathway (KP) participates in the immunoregulation through a variety of mechanisms solely or with the synergy of sCD83, and the imbalances of metabolites of KP were associated with immune dysfunction. With the complement of sCD83 to IDO-KP, a previously known immunomodulatory axis, this review focused on an expanded neuroimmunomodulation axis: sCD83-IDO-KP and its involvement in nervous system diseases.

## Introduction

### sCD83 and Immunomodulation

CD83 is a type I transmembrane glycoprotein belonging to the immunoglobulin superfamily members (1), which is mainly expressed on membrane of mature dendritic cells (DCs) of human or mice (2), and considered as a specific marker of DCs. In addition to DCs, CD83 can be expressed on activated T cells (3), B cells, macrophages, and certain brain cells. There are two forms of CD83: membrane-bound CD83 (mCD83) is presented on the surface of the mature DCs membrane, soluble CD83 (sCD83) is released from the DCs membrane and dissolved into the body fluids.

sCD83 was found in healthy human serum and confirmed to possess immunosuppressive properties (4). Staab et al. established effective sCD83 expression and purification regimens with eukaryotic human embryonic kidney 293T cells (5). Guo et al. isolated and purified sCD83 molecules from *Pichia pastoris* (1). sCD83 inhibited the differentiation process of monocytes into DCs *in vitro* and during which there was a feedback regulatory mechanism (6). sCD83 also inhibited the maturation of T cells and immature DCs stimulated by mature DCs (7) and the expression and release of CD83 from mature DC membranes induced by lipopolysaccharide. Moreover, the production of autoreactive antibodies was confirmed to be regulated by sCD83 (8). The expression of indoleamine 2,3-dioxygenase (IDO), the rate-limiting enzyme in kynurenine pathway (KP) of tryptophan metabolism, was identified as the major molecular mechanism associated with the protective effects of sCD83 (9). Mediated by IDO and transforming growth factor-β (TGF-β), the immunomodulatory effects of sCD83 were associated with CD4^+^CD25^+^Foxp3^+^ regulatory T cells (Tregs). Studies *in vitro* had shown that sCD83 induced long-term expression of IDO in DCs through autocrine or paracrine of TGF-β, whereas the latter was an essential cytokine for IDO-dependent immune tolerance (10).

sCD83 was involved in the pathogenesis of immune-related diseases, such as multiple sclerosis and its animal models of experimental autoimmune encephalomyelitis (EAE) (11), systemic lupus erythematosus (SLE) (8), transplant rejection (12, 13) and would probably provide promising approaches for the treatment of autoimmune diseases.

### IDO and Immunomodulation

Indoleamine-2,3-dioxygenase, an enzyme containing heme in the cytoplasm, is widely expressed in a variety of mammalian tissue cells, such as endothelial cells, macrophages, microglia, monocytes, DCs, fibroblasts, and certain cancer cells (14, 15). As the only extrahepatic rate-limiting enzyme that catalyzed the oxidative cleavage of indole ring structure in tryptophan (Trp) molecules along the KP (16), IDO was first identified in the intestinal tissue of rabbits (17). IDO expression is mainly distributed in the thymic medulla and the T cell regions of secondary lymphoid organs and dispersedly seen in immune tolerance or immunologically privileged sites such as the placenta, gastrointestinal mucosa, epididymis, anterior chamber, and brain tissue. By catabolizing Trp, cells expressing IDO induced the production of kynurenine metabolites, which orchestrated local and systemic responses to control inflammation, thus maintaining immune privilege (18).

Compared with normal condition, IDO expression in a variety of pathological processes increased significantly. IDO played an important metabolic immunoregulatory role through diverse mechanisms in tumor immune escape (19), maternal–fetal tolerance (20), chronic inflammatory diseases (21), autoimmune diseases (22), and transplantation tolerance (23). Trp is essential for T cell activation and hyperplasia. The induction of IDO by interferon-γ (IFN-γ) leaded to depletion of Trp in co-culture of monocytes and serum (24), which resulted in T cell proliferation arresting at Gl phase and reactivation disorder, thus bringing about the lack of effector T cells (25). With significant cytotoxic effect, l-kynurenine, picolinic acid (PIC), and other Trp catabolic products directly inhibited the proliferation of T cells and induced T cell apoptosis. IDO played immunoregulatory roles with the synergy of Tregs. IDO inhibited the activation of T cells by inducing proliferation of Tregs, resulting in the formation of local immune tolerance. Tregs promoted the production of IFN-γ, which increased the expression of IDO, and the latter strengthened the immune regulation of Tregs through feedback effects (26). Through the positive effects of DCs, Tregs directly promoted self-formation from helper T cells (27). With increased IDO expression upon IFN-γ stimulation *in vitro* and *in vivo*, microglial cells were able to block T cell responses by both Trp depletion and induction of Tregs (28). IDO was the mediator of communication between immunomodulation and oxidative stress (29). Activation of IDO-mediated tryptophan metabolism was strongly redox-sensitive and was involved in a variety of immunological diseases (30, 31). In pathological conditions, IDO mediates apoptosis through various mechanisms. By expressing abundant IDO, human umbilical cord-derived mesenchymal stem cells induced T lymphocyte apoptosis with significantly increased expression of caspase 3 (32). 1-Methyl-tryptophan (1-MT), an IDO inhibitor, promoted the apoptosis of hepatic stellate cells by increasing the expression of IFN-γRβ, IRF-1, and FAS (33). Blocking the IDO/aryl hydrocarbon receptor (AhR) metabolic circuitry resulted in enhanced repression of tumor growth by apoptosis of tumor-repopulating cells (34).

### Agonists and Inhibitors of IDO

**Figure 1:**
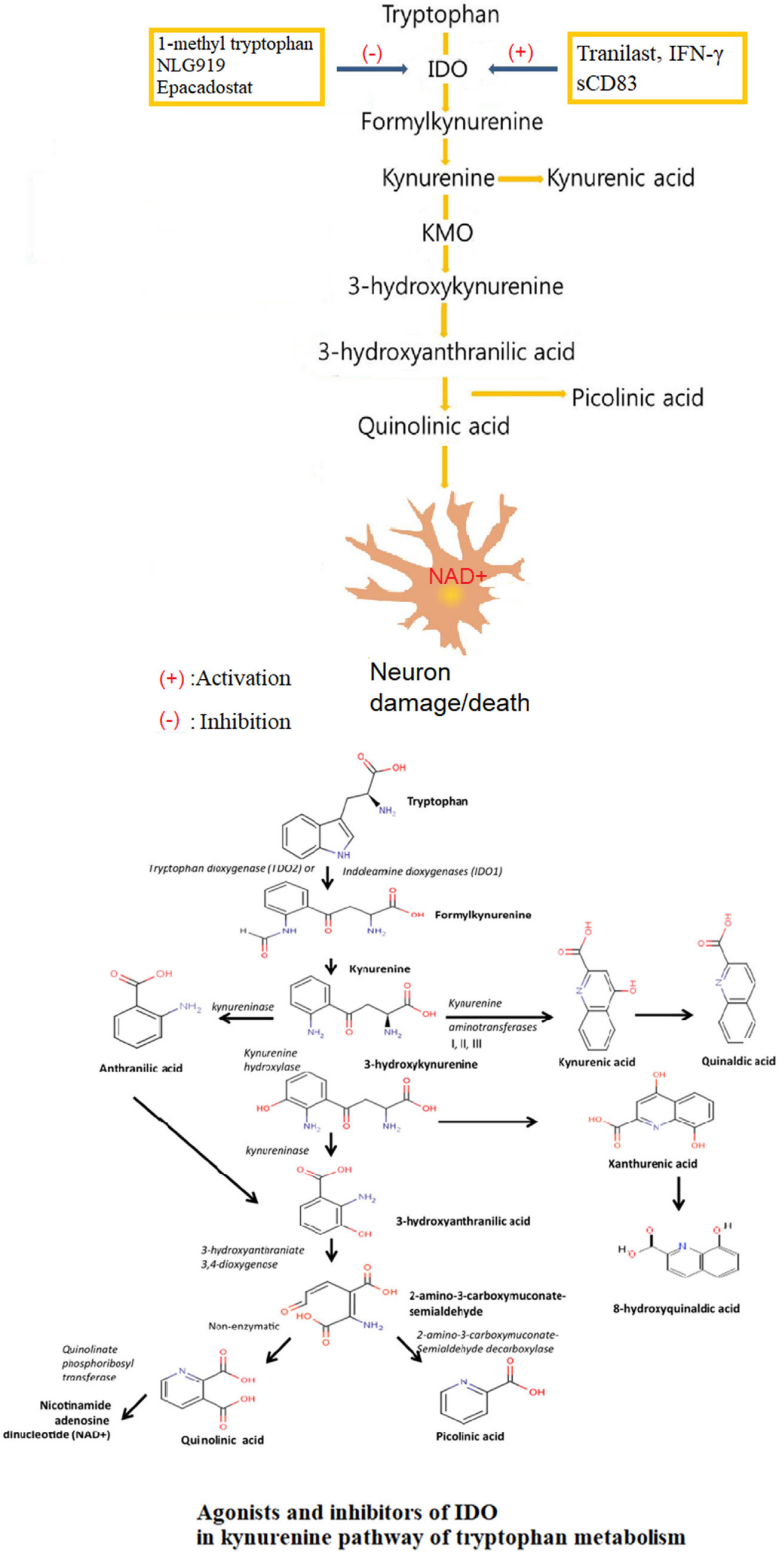
Agonists and inhibitors of indoleamine 2,3-dioxygenase (IDO) in kynurenine pathway (KP) of tryptophan metabolism. Tryptophan is finally converted to nicotinamide adenine dinucleotide (NAD+) through a series of biochemical steps along the KP with IDO as the rate-limiting enzyme and many neuroactive intermediates. The neuroprotectants include kynurenic acid and picolinic, and the neurotoxin, mainly QUIN. The role of IDO in KP is under tight control of a variety of factors, with tranilast, IFN-γ and sCD83 as agonists, and 1-methyl tryptophan (1-MT), NLG919, Epacadostat as inhibitors.

The immunoregulatory role of IDO in KP of tryptophan metabolism is under the tight control of a variety of factors (35) [Figure 1, Lee et al. (36)]. At the same time that IDO was separated and determined in 1967, researchers found that its native substrate, l-tryptophan, could inhibit the activity of IDO at high concentrations (37). Administration of a large dose of Trp could not significantly enhance IDO activity in rat intestine, which might be explained in part by a possible substrate inhibition mechanism (38). The inhibition of IDO activity with l-tryptophan loading exerted protective effects by suppressing the formation of neurotoxic substances and nitric oxide synthase in many central nervous system (CNS) disorders (39).

IDO is an enzyme that causes immunosuppression in tumors. In humans, elevated IDO expression in colon and ovarian cancer is associated with a poorer prognosis. Pharmacological, genetic, and immunological methods targeting IDO showed therapeutic benefits (40), and several pharmacological IDO inhibitors were undergoing clinical evaluation (41). Studies confirmed that 1-methyl tryptophan (1-MT) and other tryptophan structural analogs had certain IDO inhibitory activity (42). 1-MT was regarded as a specific inhibitor of IDO (43). NLG919, a newly developed IDO inhibitor, was effective in inhibiting IDO-induced T cell suppression (44). Studies *in vitro* confirmed that NLG919 promoted tumor tissue atrophy, which was further strengthened by d-1-methyl-tryptophan (D-1-MT) (45). With the significant pharmacological effects, NLG919 had entered phase I clinical trial but with a result of failure. Indoximod (d-1-methyl-tryptophan), a competitive inhibitor of IDO, can induce tumor responses in individuals with metastatic solid tumors (46). Indoximod functions as a tryptophan mimetic that suppresses the downstream effects of IDO activation on amino acid-sensing pathways and mammalian target of rapamycin (mTOR) signaling. Preclinical data support the ability of indoximod to reverse IDO-mediated immune suppression. D-1-MT treatment can reactivate mTOR suppressed by IDO1-mediated Trp depletion *in vitro*. Epacadostat (INCB024360), a potent and selective IDO enzymatic inhibitor, acted through affecting the downstream metabolites of KP, and a phase 1 dose-escalation study had been initiated in patients with advanced solid tumors (47).

There was an IFN-γ-IDO axis for IDO expression regulation and related immunoregulatory effects in different cell species. As a principal effector, IFN-γ most potently stimulated the expression of IDO (48). IFN-γ induced the gene expression and enhanced the enzyme activity of IDO (49).

N-(3,4-dimethoxycinnamonyl) anthranilic acid (3,4-DAA), known as tranilast in pharmacopeia, was a synthetic anthranilic acid derivative. With anthranilic acid as the core structure, Tranilast shared similar chemical structure with a variety of intermediate metabolites of tryptophan in KP mediated by IDO (50). Researches *in vivo* confirmed that tranilast upregulated the expression of IDO as an IDO-specific agonist (51). Tranilast had been approved in Japan and South Korea for the treatment of bronchial asthma, atopic dermatitis, allergic conjunctivitis, and scar hyperplasia (52, 53). Subsequent studies indicated that tranilast reversed paralysis in mice with EAE, offering a new strategy for treating TH1-mediated autoimmune disease such as multiple sclerosis (51).

### Kynurenine Pathway

Trp is an essential amino acid that cannot be synthesized by the human body and must be obtained from the diet. Only Trp in free form could be transported across the blood–brain barrier (BBB) by non-specific L-type amino acid transporter (54). In the CNS, Trp acted as a precursor in several metabolic pathways, such as for the production of kynurenine (Kyn), serotonin, and melatonin (55), in addition to its role in protein synthesis. In human body, the vast majority of Trp, accounting for 95% of all, was metabolized directing to production of kynurenines (56), which was called KP (57) [Figure 1, Lee et al. (36)]. IDO was the rate-limiting enzyme of KP founded in various cells, including macrophages, microglia, neurons, and astrocytes. The KP presented in most cell types of the CNS, including astrocytes (58), neurons (59), infiltrating macrophages and microglia, oligodendrocytes (60), and endothelial cells (61). The extrahepatic KP became more significant under conditions of immune activation (62). The first and rate-limiting step of the KP was the oxidative cleavage of the 2,3-double bond of the indole ring of L-tryptophan, catalyzed by the iron dependent dioxygenases (63).

The biologically active metabolites of KP mainly included Kyn and its decomposition products such as neuroprotective kynurenic acid (KYNA) (64), neurotoxic 3-hydroxy-L-kynurenine (3-OH-KYN) and its downstream enzyme metabolites, 3-hydroxyanthranilic acid (3HAA) (65), the excitotoxin, and *N*-methyl-d-aspartate (NMDA) receptor agonist, quinolinic acid (QUIN) (66), PIC (67), and the final product nicotinamide adenine dinucleotide (phosphate) [NAD+ (P+)] or NAD(P)H (68, 69). KYNA was a NMDA receptor antagonist as well as a non-competitive antagonist of the nicotinic receptor α-A7 subtype (70). QUIN, 3-OH-L-KYN, and 3-OH-AA were responsible for the production of the extremely reactive free radical species. The central intermediate of the KP was L-kynurenine (L-KYN), where the metabolic pathway divided into two different branches. L-KYN was transformed to either KYNA or 3-OH-KYN, which was further metabolized in a sequence of enzymatic steps to yield finally NAD that played essential roles in several biological processes such as redox reactions essential for mitochondrial function (71).

**Figure 2:**
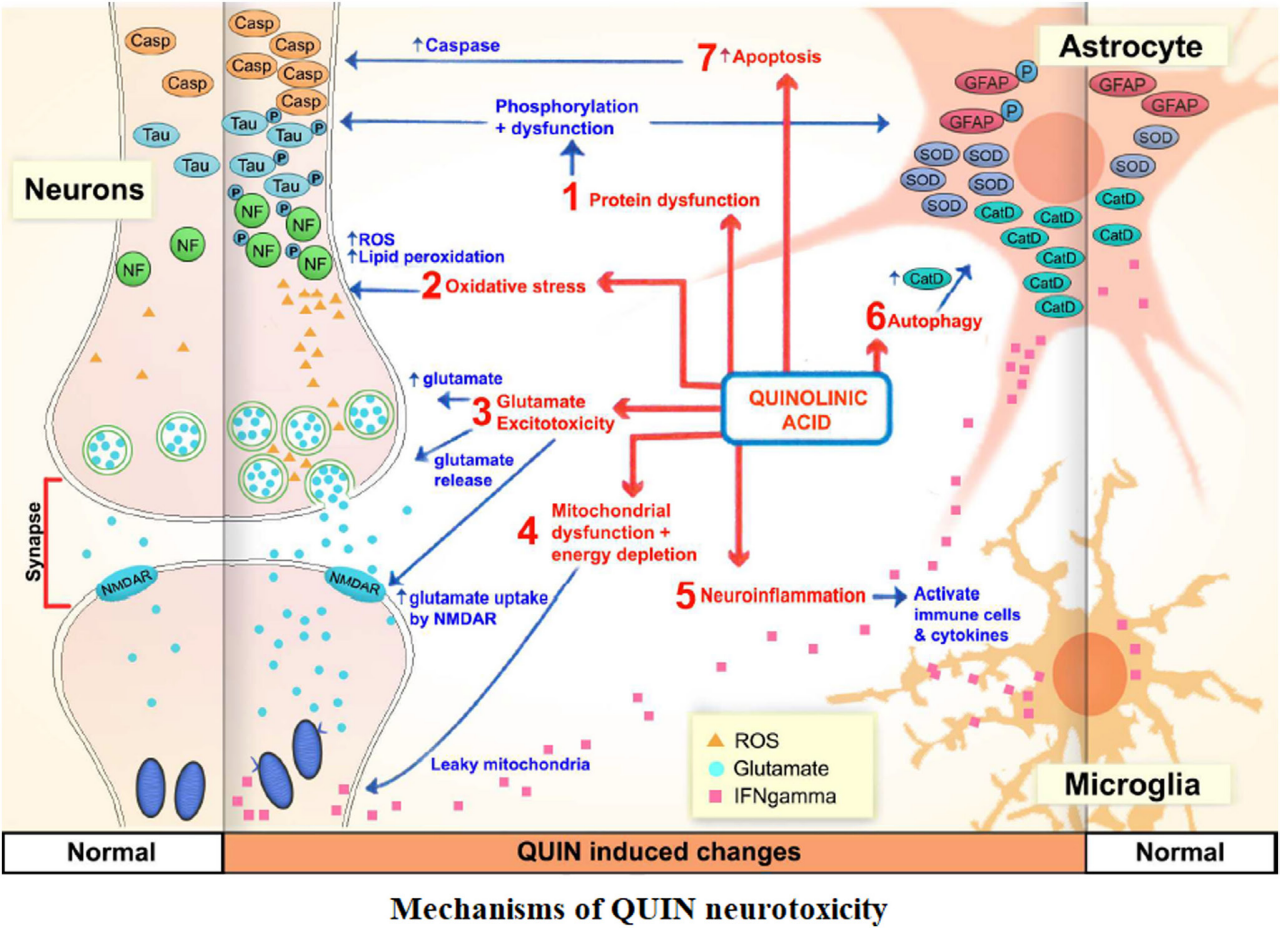
The mechanisms of QUIN neurotoxicity. Increased QUIN expression levels could increase blood–brain barrier permeability, initiate and/or exacerbate a myriad of neurotoxic processes, such as mitochondrial dysfunction, excitotoxicity, oxidative stress, protein phosphorylation, and autophagic processes.

QUIN was possibly the most important metabolite of KP in terms of neurobiological activity with a significant increase following inflammation and immune activation (72). QUIN disrupted the integrity of the BBB (73). Neurons within the neocortex, striatum, and hippocampus were sensitive to QUIN, but cerebellar and spinal cord neurons were insensitive (74). QUIN leaded to chronic dysfunction or death of human neuron in a concentration dependent manner through different mechanisms, including protein dysfunction (75), oxidative stress (76), glutamate excitotoxicity (77), mitochondrial dysfunction and energy depletion (78), neuroinflammation (79), autophagy (80), and apoptosis (81) [Figure 2, Lee et al. (36)].

Under pathological conditions, the concentrations of l-tryptophan, vasoactive l-kynurenine, neuroactive KYNA, QUIN, 3-OH-L-KYN, and enzymes responsible for their formation were significantly changed in blood, urine as well as in the brain (82). These Trp metabolites and related enzymes played short-term antimicrobial and long-term immunosuppressive roles (83). Certain Kyn productions from astrocytes played a protective role against neuronal excitotoxic-induced cell death (84). The KP contributed to immunosuppression mainly through the following four mechanisms: Trp depletion and the suppression of mammalian target of rapamycin 1 pathway, a process inhibiting effector T cell (T-eff) function and growth (85); the direct effect of Kyn on the AhR, inducing a predominant immune suppression through Tregs, especially in chronic inflammation (86); promoting the differentiation of CD4 T cells into Tregs expressing cytotoxic T lymphocyte antigen-4 (CTLA-4) and *via* phosphatase and tensin homolog encoded by a tumor suppressor gene (87); the Kyn-mediated inhibition of IL-2 signaling, which impaired memory CD4 T cell survival (88). Additionally, some of the kynurenines selectively targeted immune cells undergoing activation, resulting in the suppression of T cell proliferation (36). In conjunction with the adenosine/purinergic pathway and immune checkpoints such as CTLA-4 and programmed cell death-1 (89), KP contributed to immune privilege within organs.

With important neurological activity, the KP metabolites exerted neuroprotective or neurotoxic effects by affecting the brain glutamate, cholinergic and dopaminergic neurotransmitter delivery systems (90). Alterations in the KP had clinical and therapeutic implications, and targeting various KP enzymes was a possible strategy for addressing a variety of immune, cognitive, and neurodegenerative diseases (91, 92).

Previous studies have confirmed that both sCD83 and IDO can independently play immunoregulatory roles. Metabolites in KP of tryptophan metabolism also contribute to immunoregulation through cellular or molecular mechanisms in many neuroimmunological diseases. With the confirmation of IDO expression as the main molecular mechanism of the protective effects of CD83, and given the role of IDO as the rate-limiting enzyme in KP, an expanded immunomodulatory axis, sCD83-IDO-KP and its downstream metabolites, is formed, which may provide a more broader perspective for pathogenesis study and more target choices for drug intervention researches of immunological diseases.

## KP in Neurologic Diseases

### KP in Ischemic Stroke

Ischemic stroke is one of the major disabling and lethal diseases in adults. The pathogenesis of ischemic stroke is the decrease or complete disruption of local cerebral blood flow leading to ischemia and hypoxia, abnormal energy metabolism, cell dysfunction, and tissue necrosis.

Being enhanced both in animal models and patients with acute cerebral infarction, KP activity was significantly associated with severity and prognosis of cerebral infarction (93). Inflammation in cerebral infarction activated KP and induced the expression of IDO (94). Changes in kynurenine metabolites of KP had been confirmed to correlate with the infarct volume and the mortality of stroke patients (95). Earlier studies showed that elevated levels of QUIN after cerebral infarction were associated with reduced nerve cells in different regions of the brain. KYN expression in patients with blood stasis cerebral infarction was significantly higher (96). Studies about L-KYN in rodent stroke models showed conflicting results, the reason for which at present is unclear. l-kynurenine sulfate was protective when issued prior to induction of cerebral ischemia (97). In post-ischemic treatment, l-kynurenine sulfate worsened the histopathological outcome of a mouse stroke model. Deteriorated histopathological changes were observed in a focal ischemia/reperfusion model with distal middle cerebral artery occlusion treated with L-KYN, indicating that post-ischemic treatment with L-KYN might be harmful (98). As an endogenous ligand that mediated AhR activation in the brain after occlusion of the middle cerebral artery, L-KYN inhibited the combination of cyclic adenosine monophosphate response element with protein-dependent signaling that leading to acute brain injury after stroke (99). The inhibition of kynurenine 3-monooxygenase (KMO) activity reduced brain injury in experimental models of ischemic stroke. 3-hydroxyhydroanthranilic acid (3-HAA) and anthranilic acid (AA), downstream metabolites of KYN, were directly related to the volume of cerebral infarction. An synthetic KYNA analog, N-(2-N,N-dimethylaminoethyl)-4-oxo-1H-quinoline-2-carboxamide hydrochloride exerted neuroprotective effects in animal cerebral ischemia models because of the NMDA antagonism effects.

### KP in Epilepsy

Epilepsy is a clinical syndrome that characterized by paroxysmal CNS dysfunction, caused by recurrently and suddenly abnormal discharge of brain neurons and manifested as paroxysmal motor sensory abnormalities, autonomic dysfunction, consciousness, and mental state changes.

Neuroactive metabolites of KP with amino acid structures played an important role in epileptic pathophysiology. The incidence of epilepsy was associated with decreased levels of KYN and KYNA in brains of the patients and increased production of KYNA relieved the symptoms of drug-related epilepsy. The changes of KP enzyme activity which resulted in the imbalance between glutamic acid (GLU)/gamma-aminobutyric acid (GABA) in epileptic foci were involved in the pathogenesis of epilepsy (100).

Intravenous injection of QUIN or its direct injection into specific parts of the experimental animal brain could lead to epilepsy-like seizures in rats. Researches confirmed that particular anatomical site of the hippocampus was sensitive to QUIN excitotoxicity (101). In gliocytes proliferating in epileptic foci, the levels of QUIN synthetase, 3-hydroxyanthranilate oxidase, were relatively increased; the activity of quinolinate phosphoribosyltransferase (QPRTase), a QUIN degrading enzyme, decreased. PIC enhanced epilepsy activity and was essential for the pathogenesis of drug-resistant epilepsy (102). As naturally occurring vesicular glutamate transporters (VGLUTs) inhibitors, Kynurenine and xanthurenic acid inhibited the uptake of glutamate by presynaptic membrane into vesicles, thereby affecting synaptic function. VGLUTs dysfunction was involved in many neuropsychiatric disorders such as epilepsy and schizophrenia (103). KP metabolism of Trp provided a reasonable explanation for the correlation between epilepsy and depression. Neurobiochemistry and behavioral studies found that minocycline, an IDO inhibitor, could promote antiepileptic effects of valproate in a dose-dependent manner, and improve depression associated with epilepsy (104), Quercetin combined with levetiracetam had a similar effect (105).

### KP in Alzheimer’s Disease (AD)

Alzheimer’s disease is one of the most prevalent progressive neurodegenerative diseases, the most common type of dementia with insidious onset mainly affecting the elderly (106). The characteristic clinical manifestations include the deterioration of memory function, progressive decline in daily living capacity, various neuropsychiatric symptoms and behavioral disorders. Extracellular amyloid-β accumulation and intracellular tau deposition are the significant features of the pathomechanism related to AD (107).

Neuroendocrine disorders served as one of the pathogenesis of AD (108). Glutamate excitotoxicity, neuroinflammation, cerebrovascular dysfunctions, and mitochondrial metabolic disorders were also factors known to contribute to the neurodegenerative process (109). The enhanced IDO expression and increased KYN/TRP ratio reflecting the activity of KP in tryptophan metabolism were observed in serum of AD patients, exhibiting an inverse correlation with the cognitive decline (110). Imbalances in the KP had been observed in many disorders with cognitive decline including AD, and influencing this delicate balance might be of therapeutic value (111).

Induced by upregulated generation of IDO mediated by interferon (IFN)-gamma and other proinflammatory cytokines, the production of various neurotoxic substances of KP involved in the pathogenesis of AD were increased (112, 113). QUIN was particularly associated with the pathogenesis of AD (59). Studies confirmed that QUIN immunoreactivity in cerebral hippocampus of AD patients was related to disease progression. Increased levels of QUIN were observed in both the cerebrospinal fluid (CSF) and serum of AD patients, causing significant neuronal damage either by direct activation of NMDA receptors or the release of endogenous glutamate (114). QUIN promoted the expression of IDO in small keratinocytes and astrocytes, which in turn affected KP. In brains of AD patients, QUIN accelerated cell damage by oxidative stress, aggravated the pathological changes as an inducer of β-amyloid peptide and promoted the release of extracellular glutamate which could directly cause neurotoxicity. QUIN was not only co-localized with hyperphosphorylated tau in the AD cortex, but also capable of inducing tau phosphorylation in primary neurone cultures (115). KYN-NAD was involved in AD since Aβ 1-42, a cleavage product of amyloid precursor protein, induced the production of QUIN with neurotoxic concentrations from macrophages and microglia (116).

Kynurenic acid levels were altered in the brain and CSF of AD patients with its significance differently described in various research conclusions (117). In brains of pathologically confirmed AD patients, decreased levels of L-KYN, 3-OH-KYN, and elevated activity of KYNA and kynurenine aminotransferase-I (KAT-I) had been detected in the striatum and caudate nucleus (118). Kling et al. showed that KYNA had a positive impact on AD by promoting the activity and gene expression of enkephalinaseand and facilitating Aβ degradation (119). KYNA exerted its neuroprotective effects by promoting amyloid degradation and increasing the neuronal cell survival through induction of the gene expression of neprilysin (120). Other mechanisms involved in the neuroprotective effects of KYNA and its derivatives included preferentially inhibiting of extrasynaptic NMDA and α7 nicotinic acetylcholine receptors while sparing the synaptic NMDA-mediated currents (121).

The level of 3-hydroxy-kynurenine (3-HK), a downstream metabolite of tryptophan in KP, increased in the brain and serum of AD patients (122). Through increasing β-amyloid protein accumulation, glial activation, oxidative stress and positive feedback regulation mechanism in KP, 3-HK exacerbated the neurodegenerative lesions of AD. 3-HK inhibited the activity of mitochondrial respiratory chain enzyme complex I, II, III, resulting in energy metabolism disorder in neurons and subsequent nerve tissue injuries.

Studies indicated that blocking KYN pathway could protect mouse models of AD. The application of KP enzyme inhibitors or its metabolite analogs had been shown to be effective in the treatment of AD in preclinical studies. The extracts of rhizoma coptidis demonstrated therapeutic effects on AD through inhibiting the activity of microglia and astrocytes and the expression of IDO, blocking the loss of neurons and reducing the formation of amyloid plaques in AβPP/PSI transgenic mice (123). Administered together with a transport inhibitor of KYNA known as probenecid, L-KYN prevented the morphological alteration and cellular damage caused by soluble Aβ which further resulted in a significant improvement of the spatial memory (124).

### KP in Multiple Sclerosis

Multiple sclerosis (MS) is a demyelinating disease of the CNS, which is also regarded as a neurodegenerative disease triggered by inflammation. Multiple demyelinating plaques in the CNS white matter are the pathological characteristics of MS. Demyelination may partially or wholly involve the white matter of the lateral ventricles, optic nerve, spinal cord, cerebellum, and the brain stem, among which, periventricular lesions are especially typical. Demyelination founded in the cerebral cortex of MS patients might be the cause of cognitive impairment (125). The pathogeny of MS has not been elucidated. The particular effects of various KP metabolites on nervous system (126), imbalance between neuroprotective and neurotoxic intermediates of KP and immunomodulatory effects mediated by IDO were all involved in the pathogenesis of MS (127). Recent studies suggest that abnormalities in the KP may be associated with the switch from early mild stage MS to debilitating progressive forms of MS and that analysis of KP metabolites in MS patient serum may have application as MS disease biomarkers (128).

Associated with disease condition, there was activation of the KP in MS. Decreased levels of Trp in serum and CSF of MS patients suggested an increase in KP activity, the increased serum L-KYN levels in MS patients treated with IFN-β were associated with disease remission (129). KYNA is the only known endogenous ionotropic glutamate receptor antagonist that inhibits presynaptic α7 nicotinic acetylcholine receptors, regulates presynaptic glutamate release. Changed with patient’s condition, the fluctuation of KYNA levels in CSF demonstrated the probable neuroprotective effects on MS. QUIN was a potent agonist of NMDA receptors that could promote the onset of MS through inducing glutamate excitotoxicity, lipid peroxidation, and oxidative stress injury (130). Elevated levels of QUIN in the spinal cord of EAE rats were associated with an acute disease course (131). Multiple studies had consistently found structural changes in a variety of cellular proteins associated with QUIN that leading to the death of oligodendrocytes, neurons, and astrocytes. The QUIN/KYNA ratio has strong correlation with the disability and severity of MS patients (128). 3-HK, another potential neurotoxin, was found to be significantly higher in the serum of MS patients (128). QUIN and 3-HAA analogs selectively leaded to the apoptosis of Th1 cells, thereby further inducing immune tolerance. Increased levels of 3-HK in the spinal cord of EAE rats distinctly raised the production of free radicals.

Indoleamine 2,3-dioxygenase gene expression and activity were predictive of relapse in MS patients, indicating that KP orchestrated self-protective mechanisms that inhibited antigen-specific immune responses in the CNS (132). Studies confirmed that immunoregulatory effects of IDO were involved in the pathogenesis of EAE. Enhanced inflammatory responses of Th1/Th17 exacerbated EAE in IDO-deficient mice (22). IDO activation increased the level of KYNA, thereby inhibiting the proliferation of reactive T lymphocytes. The administration of 1-methyl-tryptophan (1-MT), a specific IDO inhibitor, deteriorated the condition of EAE (133).

Immunomodulatory effects mediated by KP and IDO were crucial to the pathogenesis and treatment of MS (134). Inhibitors of KP enzymes, NMDA antagonist KYNA, and its pharmacological derivatives provided new options for MS treatment (135). Enzymes and metabolites related to KP had become potential therapeutic targets for MS (16), such as endogenous tryptophan metabolites and their structural analogs (cinnabarinic acid, tranilast), IDO inhibitors (1-MT and berberine). The administration of 3HAA, a downstream KP metabolite, reduced EAE severity through increasing the proportion of Tregs and inhibiting Th1/Th17 response. Tranilast was an oral active synthetic Trp metabolite that inhibited myelin-specific T cell proliferation and the release of proinflammatory cytokines from Th1 cells, relieved inflammatory responses, modulated immunosuppressive effects, thereby reducing disease relapse frequency and the disease severity during relapse. Sundaram et al. (136) found that IDO inhibitors significantly reduced the levels of QUIN in cultured cells and eliminated oligodendrocyte apoptosis. As an endogenous Trp metabolite, cinnabarinic acid was a partial agonist of metabotropic glutamate receptor 4 (mGluR4), which has a protective effect on EAE. Treated mGlu4-deficient mice with cinnabarinic acid caused a turning of immune response to Tregs (137).

### KP in Vascular Cognitive Impairment (VCI)

Vascular cognitive impairment is defined as a syndrome with evidence of clinical stroke or subclinical vascular brain injury and cognitive impairment affecting at least one cognitive domain, including attention, memory, language, visuospatial skills, and abstract reasoning (138, 139). Microvascular inflammation, cerebrovascular dysfunction, and neurodegeneration were all involved in the development of VCI as pathogenic mechanisms (2, 140).

Previous studies described an IFN-γ-IDO-KYN and inducible nitric oxide synthase (iNOS) pathway through which microvascular immune inflammation resulted in VCI. The impact of chronic inflammation on VCI was mediated by the unique ability of IFN-γ to transcriptionally induce the rate-limiting enzymes of tryptophan–kynurenine metabolic pathways with the synergistic action of tumor necrosis factor-α (TNF-α) (141). Triggered by upregulated production of IFN-γ in periphery (macrophages) and brain (glia), a merger of tryptophan–KP into inflammation cascade involved in VCI (142). IFN-γ-induced upregulation of IDO increased the levels of certain metabolites of KYN associated with cognitive impairment. The significantly increased levels of iNOS in CSF of patients with vascular dementia (VD) were involved in VCI by mediating excitatory neurotransmission and affecting synaptic plasticity. Under pathological conditions, excessive release of NO induced by positive feedback loop in inflammation that mediated by IDO leaded to neuronal oxidative stress injury which affected the learning and memory function of brain (16).

Indoleamine 2,3-dioxygenase and KYN metabolites increased markedly with deterioration of cognitive impairment accompanied with immune activation and nerve injury (8). The long-term activation of the KP resulted in an imbalance in critical neuroactive compounds involving the reduction of tryptophan and increase of tryptophan metabolites, which had definite implications in cognitive impairment (143). KP metabolites, some kynurenines (e.g., quinolinic and PICs) had also been implicated in VCI (55). Changes of kynurenine metabolites had also been suggested to correlate with the post-stroke cognitive impairment. Activation of IDO and increased production of kynurenine metabolites had been observed in post-stroke cognitive impairment patients. One study demonstrated an association between elevated kynurenine/tryptophan (K/T) ratios and the extent of cognitive impairment in patients with acute ischemic stroke, and what is more, the K/T ratio was described as an important biomarker of VCI (144). Kynurenine was also identified as candidate diagnostic biomarker for VCI in a metabolomics study, which provided a novel strategy for stratifying stroke patients with cognitive impairment using serum-based metabolite markers and might be of great importance in further investigating the pathological mechanisms of VCI (145).

VCI was also identified as age-associated neuroendocrine disorders. The transcriptional induction and activation of IDO shifted tryptophan metabolism from serotonin synthesis to formation of “kynurenines,” which might contribute to development of VCI *via* their apoptotic, neurotoxic, pro-oxidative effects, and the upregulation of iNOS, phospholipase A2, arachidonic acid, prostaglandin, 5-lipoxygenase, and leukotriene cascade (146).

### KP in Depression

Depression is commonly characterized by anhedonia (loss of interest or pleasure) for 2 weeks or longer and the presence of at least four of the following persistent symptoms: weight loss or gain, sleep disturbances, psychomotor agitation or retardation, fatigue, worthlessness or inappropriate guilt, diminished concentration, or indecisiveness (147). As one of the most common mental disorders and social issues worldwide, depression causes a low quality of life or even suicide for many people (148). However, the underlying mechanisms of depression remain elusive and the effectiveness of the currently used antidepressants is still far from satisfactory (149).

The hypothesis of cytokine-induced sickness behavior, which was recognized within a few years of the cloning and expression of interferon-alpha, IL-1 and IL-2, may provide some meaningful references. The proinflammatory cytokines activated in the brain can induce common symptoms of sickness, such as loss of appetite, sleepiness, withdrawal from normal social activities, and fatigue. These cytokine actions may offer some of the first clues about the pathophysiology of certain mental health disorders, including depression.

Tryptophan degradation and its role in the availability of serotonin and the setting of inflammation had brought attention to the KP for potential mechanism and treatment studies of depression (150). Kynurenine metabolism was hypothesized to be a key mechanism that linked inflammation and depression. Increased inflammation and toxic KP activation, including excitotoxicity mediated by NMDA receptor, as well as decreased 5-HT levels were associated with pathophysiology of depression (151, 152). Bay-Richter et al. (153) revealed that levels of QUIN increased and KYNA decreased over time in suicidal depression patients versus healthy controls (153). The activation of KP as a marker of inflammation presented in a subgroup of individuals with major depressive disorder (MDD). The abnormalities in the concentrations of KP metabolites (KYNA and QUIN) were associated with reduced volume of striatum, which played the central role of in motivated behavior, reward processing, and anhedonia. The KYN/TRP ratio was inversely associated with striatal volumes in the MDD sample (154). Research revealed that relative neurotoxic shift in the balance of kynurenine metabolites with reduced KYNA/quinolinic acid (QA) was associated with sleep disturbance in the currently depressed patients. It was considered that altered kynurenine metabolism might also molecularly link sleep disturbance and depression (155).

Depression is predominately female disease but with unclear reason. With different disease forms such as premenstrual depression, postnatal depression, and perimenopausal depression, depression is obviously related to hormonal fluctuations. The successful treatment of certain common types of depression by estrogens provided further evidence. Meier et al. found that a reduction in KynA concentrations in women might constitute a vulnerability factor that partly explained the higher incidence of depression in females. Furthermore, the significant association between oral contraceptive (OC) use and reduced KynA could conceivably partially account for the epidemiological association between OC use and depression in females (156). Characterized by low tryptophan levels, increased breakdown toward kynurenine and a downstream shift toward the 3-OH-KYN arm, women in the first 2 months of the physiological postpartum period were at very high risk for the first onset of acute and severe depression (157). de Bie et al. found that progesterone attenuated interferon-γ-induced KP activity in macrophages with increased of neuroprotective KYNA levels and reduced the inflammatory marker neopterin, which could partly explain how hormones were involved in the pathogenesis of depression (158).

Patients with various chronic diseases of different systems might suffer from depression. Depression occurred more frequently in patients after stroke than in the general population (159), with a varied incidence from 18 to 61%. Several studies reported an increase activation of both tryptophan-2, 3-Dioxygenase (TDO)/IDO and also kynurenine aminotransferases, which leaded to an augmentation of 3-HK, QUIN, L-Kyn, and KYNA production, which finally induced ROS generation and alterations in glutamatergic neurotransmission in stroke patients (160). A reduction in 5-HT production and an increased KP catabolism was clearly observed in poststroke depression. Neuropsychiatric symptoms are among the earliest manifestations of SLE. Depression-like behavior was evident in the murine model of lupus. Increased levels of KP metabolites (kynurenine, 3-hydroxy-kynurenine, 3-hydroxynthranilic acid, and QUIN) in cortex and hippocampus of these murine models suggested that activation of the KP might among the potential pathophysiological mechanisms responsible for neuropsychiatric symptoms of SLE (NP-SLE) (161). Positive correlations were drawn between KYN, QUIN, KYNA, and specific pro-inflammatory immunological variables in the CSF, and depressive symptoms in patients with hepatitis on IFN-α treatment. In addition, a significant relationship was found between depression developed in patients with chronic viral hepatitis after IFN therapy and especially the type of IFN-α (162). With significantly higher kynurenine levels and KYN/TRP ratios post-chemotherapy, many women with breast cancer experienced more severe depression after chemotherapy treatment (163). Chronic inflammation was the likely common instigator between cardiovascular pathology and depression. Certain pro-inflammatory substances released by macrophages and microglia upregulated the rate-limiting enzymes in the KP, resulted in the formation of neurotoxic metabolites. Inflammation was closely associated with endothelial dysfunction in cardiovascular disease, a preamble to atherosclerosis and atherothrombosis, which had also been detected in depression (164).

### KP in Parkinson’s Disease (PD)

Parkinson’s disease is a chronic progressive neurodegenerative disorder characterized by loss of dopaminergic neurons in the midbrain and presence of localized neuroinflammation and protein inclusions called Lewy bodies in the midbrain several years before the actual onset of symptoms (165, 166). PD is normally identified by motor symptoms (bradykinesia, rigidity, resting tremor, and gait disturbances), which are believed to be largely related to the loss of nigral dopamine neurons. Moreover, patients also frequently exhibit a range of non-motor disturbances including constipation, hyposmia, depression, and cognitive decline (167), being more debilitating than motor signs and worsening with disease progression (168).

The detailed pathogenesis of PD is unclear, but several mechanisms have been proposed including neuroinflammation, glutamatergic neurotoxicity, the dysregulation of the KP and alterations in serotoninergic and melatoninergic pathways (35). The KP of TRP catabolism is implicated in the inflammatory and neurotoxic events in Parkinsonism. Studies demonstrated that increased TRP catabolism positively correlated to the increases of inflammatory markers (IL-6, CRP, and MCP-1) in both the periphery and CSF of PD patients, which were associated with non-motor symptoms of PD such as fatigue, depression and cognitive impairment (169). Lewitt et al. assayed CSF of pathologically verified PD subjects using ultra-high-performance liquid and gas chromatography linked to mass spectrometry. The results showed that the mean 3-HK-KYN concentration was increased and the mean oxidized glutathione was decreased, providing direct support for the involvement of the KP and excitotoxicity in development of PD (170).

Several neuroactive compounds are produced through the KP, among which KYNA and QUIN are intensively studied in PD. KYNA possessed antioxidant properties and was a non-competitive antagonist of a7-nicotinic acetylcholine receptors at physiological levels regulating the levels of acetylcholine, dopamine, and glutamate in the CNS (171). QUIN could activate the NMDA receptor-signaling pathway leading to excitotoxicity and amplify the inflammatory response (164).

The increase in KYNA not only modulated glutamate release from cortical areas to striatum but also directly acted on the NMDA receptors as antagonist, thereby limiting glutamate excitotoxicity. Ogawa et al. reported for the first time that TRP/KYN and KYNA/TRP ratios were significantly increased in the frontal cortex, putamen of PD patients (172). There were also increased Kynurenine/Tryptophan (K/T) ratios in both serum and CSF of PD patients compared to controls (173, 174). By driving tryptophan down the KP, alterations in gut microbiota were widely accepted as relevant to the etiology, course, and treatment of many neuropsychiatric disorders, including PD (175). Investigators observed altered levels of kynurenine metabolites in PD patients with L-3, 4-Dihydroxyphenylalanine (L-DOPA)-induced dyskinesia. There were obvious increases of the 3-HK-MYN/KYNA ratio and 5-hydroxytryptophan (5-HTP) levels, but decrease of anthranilic acid levels in plasma and CSF of this patient group. Through affecting the KP of tryptophan metabolism, L-DOPA might play a role in the development of L-DOPA-induced dyskinesia as the most effective drug in the symptomatic treatment of Parkinson’s disease (176).

QUIN, a metabolite of the KP of tryptophan catabolism, plays a role in the oxidative stress associated with many neurological disorders including Parkinson’s disease. Intrastriatal administration of QUIN in rats was reported to be able to induce significant behavioral changes including involuntary movements (177). Through activating NMDA receptor, endogenous QUIN released by microglia played a crucial role in mediating the progressive loss of dopaminergic neurons in PD (178). Recent researches indicated that QUIN was directly involved in mood and behavioral changes in PD patients (179). The deficits in spatial reference memory had also been observed in rats following the development of QUIN lesions, suggesting that QUIN could induce cognitive deficits in PD patients. Apart from its excitotoxic effects, QUIN could also enhance lipid peroxidation in an iron (II) dependent manner (180).

The abnormalities of enzyme activity of KP are observed in PD. Diminished immunoreactivity of KAT-I, the KP enzyme, which leaded to KYNA formation in the pars compacta of the substania nigra, was found in mouse models of PD (181). The enzyme aminocarboxymuconate-semialdehyde-decarboxylase (ACMSD) located at a key branch-point of the KP, limited the production of the neurotoxin QUIN with inflammatory properties. The missense mutation in the ACMSD gene predicted to disrupt enzyme function in typical PD individual, suggesting that this enzyme might influence PD pathogenesis (167). Vilas et al. reported a novel ACMSD mutation resulting in the change of p.Glu298Lys amino-acid in a sporadic PD patient, which was not present in neurologically normal population, suggesting that not only common genetic variability but also rare variants in ACMSD alone might increase the risk of PD (182).

Using PD animal models, the modulation of the KP by enhancing endogenous KYNA and/or decreasing QUIN production was demonstrated to be a potential therapeutic strategy for PD (183). Administration of KYNA directly into the globus pallidus internus of parkinsonian non-primate protected against 1-methyl-4-phenyl-1, 2, 3, 6-tetrahydropyridine hydrochloride (MPTP)-induced toxicity. In addition, systemic administration of exogenous KYNA showed very limited therapeutic efficacy because KYNA had poor permeability across the BBB and a very short biological half-life. KYNA injection into the brain could protect dopaminergic neurons against QUIN or NMDA-mediated excitotoxicity. Silva-Adaya et al. reported that L-kynurenine (L-KYN) pretreatment had a protective effect on locomotor asymmetry, striatal reactive gliosis, and neurodegeneration, and changes of dopamine levels in rodent PD models (184). Further study demonstrated that synthetic kynurenine analogs had neuroprotective effects on mice model of PD (185). Silva-Adaya et al. showed that co-administration of the main KYNA precursor, L-KYN and an inhibitor of organic anion transporter could increase KYNA and result in the reversal of glutamate-induced excitotoxicity in 6-hydroxydopamine (6-OHDA)-induced PD rats (184). Analogs of L-KYN and KYNA with long half-life such as 4-Cl-L-KYN, 7- Cl-KYNA and more recently 2-(2-N, N-dimethylaminoethylamine- 1-carboyl)-1H-quinolin-4-one hydrochloride had been designed to enhance their stability and pharmacological therapeutic properties (186–188). Several preclinical studies showed that activation of group II- metabotropic glutamate receptor (mGluR) and group III-mGluR were potentially important drug targets to provide both symptom relief and neuroprotection in PD implying that, apart from KYNA, these two KP metabolites might have therapeutic importance in PD (189). When administered intraventricularly with systemic L-KYN, kynureninase inhibitors elevated KYNA levels in brain and showed protective effects against QUIN-induced toxicity in PD rats. Recent work had highlighted the therapeutic potential of inhibiting two critical regulatory enzymes in KP, KMO, and TDO. In fly models of Parkinson’s disease, Breda et al. provided genetic evidence that inhibition of TDO or KMO improved locomotor performance and ameliorated shortened life span (190). Parasram revealed that phenolic compounds, a class of phytochemicals, including flavonoids and diarylheptanoids, had been shown to reduce striatal lesion size, reduce inflammation, and prevent lipid peroxidation caused by QUIN in PD and other oxidative stress related neurological disorders (191).

A growing body of evidence suggested that the KP and its metabolites of KP were involved in the pathogenesis of PD. Modification of altered KP represented important targets to prevent the progression of the underlying neurodegeneration in PD. Drugs targeting the KP are pivotal to further understanding of the therapeutic value of KP manipulation in patients with PD.

## Conclusion

Dysfunction of neuroimmunomodulation and destruction of immune homeostasis are the pathogenesis of various neurological diseases in which a variety of immune molecules or reaction pathways were involved. With the determination of immunomodulatory property of sCD83 and related mechanisms, an expanded neuroimmunomodulation axis, sCD83-IDO-KP, was formed, which had been proved to have important impacts on the development of various neurological diseases, including ischemic stroke, epilepsy, AD, multiple sclerosis, PD, and depression. Further researches are still needed on doubts about the downstream effector molecules, other neuroactive effects of these cells and molecules besides immunoregulation and the specific processes of their interaction with immune effector cells. The answers to the above mysteries will also provide more new strategies for the prevention and treatment of neurological autoimmune diseases and other immune-related diseases.

## Author Contributions

LB is responsible for literature search, data collection, and article writing. TG determines the structure of the article and content distribution. GL determines the content and is responsible for the article review.

## Conflict of Interest Statement

The authors declare that the research was conducted in the absence of any commercial or financial relationships that could be construed as a potential conflict of interest.

## Abbreviations

sCD83, soluble CD83; IDO, indoleamine 2, 3-dioxygenase; KP, kynurenine pathway; DCs, dendritic cells; Tregs, CD4^+^CD25^+^Foxp3^+^ regulatory T cells; 1-MT, 1-methyl-l-tryptophan; 3, 4-DAA, N-(3, 4-dimethoxycinnamonyl) anthranilic acid; KYN, kynurenine; KYNA, kynurenic acid; 3-OH-KYN, neurotoxic 3-hydroxy-L-kynurenine; 3HAA, 3-hydroxyanthranilic acid; NMDA, *N*-methyl-d-aspartate; LPS, lipopolysacchatide; EAE, experimental autoimmune encephalomyelitis; QUIN, quinolinic acid; PIC, picolinic acid; BBB, blood–brain barrier.
